# Pregabalin as a neuroprotective alternative to methylprednisolone in peripheral nerve regeneration: an experimental study

**DOI:** 10.1016/j.clinsp.2025.100838

**Published:** 2025-11-11

**Authors:** İrem Ateş, Esra Laloglu, Neslihan Küçün, Sevilay Ozmen, Serkan Yıldırım, Basri Pür, Duygu Köse, Maide Sena Civelek, Agah Kahramanlar, Bahar Işık, Ali Ahıskalıoğlu, İsmail Bolat

**Affiliations:** a Department of Anesthesiology and Reanimation, Faculty of Medicine, Ataturk University, Erzurum, Turkey; b Department of Medical Biochemistry, Faculty of Medicine, Ataturk University, Erzurum, Turkey; c Department of Pathology, Faculty of Medicine, Ataturk University, Erzurum, Turkey; d Department of Pathology, Faculty of Veterinary Medicine, Kyrgyz-Turkey Manas University, Bishkek, Kyrgyzstan; e Department of Orthopedics and Traumatology, Faculty of Medicine, Ataturk University, Erzurum, Turkey; f Department of Pharmacology, Faculty of Medicine, Ataturk University, Erzurum, Turkey; g Department of Anesthesiology and Reanimation, Erzurum Training and Research Hospital, Erzurum, Turkey; h Department of Emergency, Faculty of Medicine, Binali Yıldırım University, Erzincan, Turkey

**Keywords:** Peripheral nerve injuries, Pregabalin, Methylprednisolone, Neuroprotection, Nerve regeneration, Sciatic functional index, Experimental study

## Abstract

**Background:**

Peripheral nerve injuries are particularly important in terms of their frequency and complications. The purpose of this experimental study was to investigate the neuroprotective effects of pregabalin and methylprednisolone administered at different doses and in combination in an axonotmesis model and to establish whether pregabalin can represent an alternative to methylprednisolone.

**Methods:**

Fifty-six adult male Sprague-Dawley rats were assigned to control, sham (axonotmesis only), axonotmesis + pregabalin 30 mg/kg (low dose), axonotmesis + pregabalin 60 mg/kg (high dose), axsonotmezis + 1 mg/kg methylprednisolone, axonotmesis + pregabalin 30 mg/kg + 1 mg/kg methylprednisolone (low-dose combination), and axonotmesis + pregabalin 60 mg/kg + 1 mg/kg methylprednisolone (high-dose combination) groups. Following axonotmesis, the treatment groups received oral treatment once daily for one week. Sciatic functional index, electromyography, biochemical (NGF, TGF-β, CNTF, MBP), immunohistochemical, histomorphological and immunofluorescence analyses were performed to assess the neuroprotective effects of the treatments.

**Results:**

Immunohistochemical, electrophysiological, and biochemical analyses demonstrated that the separate or combined use of pregabalin and methylprednisolone may have positive effects on nerve regeneration.

**Conclusion:**

Low-dose pregabalin and methylprednisolone alone exhibited similar effects on peripheral nerve regeneration and functional healing. Pregabalin may represent an alternative to steroids due to its fewer side effects. Although direct pain behavior assessment was not performed in this study, the effect of pregabalin on neuropathic pain may be additionally beneficial in combined use. These results demonstrate that pregabalin offers comparable neuroregenerative effects to methylprednisolone, with combination therapy proving superior. This positions pregabalin as a promising steroid-sparing agent for peripheral nerve injury, warranting further clinical investigation.

## Highlights

• Peripheral nerve injuries cause significant morbidity, necessitating better treatments.
• Low-dose pregabalin promotes nerve regeneration comparably to methylprednisolone.
• Pregabalin reduces euroinflammation (↓NF-κB) and enhances neurotrophic factors (↑NGF).
• Synergistic effect of combination therapy on functional (SFI, EMG) and histological recovery.
• Findings support pregabalin as a candidate neuroprotective agent with effects comparable to steroids.

## Introduction

The peripheral nerves can be injured and/or suffer loss of function as a result of accidents, trauma, or other causes. This can result in partial or complete loss of sensory, motor, and autonomic functions, neuropathic pain, muscle atrophy, and lifelong morbidity. Despite extensive accumulated understanding of the pathophysiology of Peripheral Nerve Injuries (PNIs) and regeneration mechanisms, reliable methods permitting complete restoration of motor and sensory function are inadequate, and research in this field is still ongoing.^1^^,^^2^ In contrast to the Central Nervous System (CNS), the Peripheral Nervous System (PNS) possesses a powerful self-repair and regeneration capacity.^3^

Axonotmesis, widely known as nerve crush injury, is one of the most frequently investigated injuries for studying nerve regeneration, and one in which the most progress has been made in recent years. In such injuries, the axon is damaged or destroyed, but much of the connective tissue is preserved. Studies have shown that the blood-brain barrier is impaired in axonotmesis injuries, but that gradual healing occurs on approximately the seventh day. Several factors are involved in the regenerative process. When peripheral nervous system axons are injured, Wallerian Degeneration (WD) commences in the distal part. A series of changes is known as WD, including nerve injury-related denaturation and breakdown of axons and myelin sheaths, Schwann Cell (SC) proliferation, macrophage and mast cell infiltration, and the removal from the lesion of necrotic cells and myelin debris. In addition to cellular responses caused by damaged axons, WD is accompanied by SC differentiation and immune response activation.^4^, ^5^, ^6^

Microphages migrate to the injury site within a few hours, and SCs proliferate. These release various neurotrophic factors that support neuronal survival, are essential for nerve regeneration, and support axon growth. During development, neurotrophic factors play a key role in nerve growth and neurodiversity in the PNS.^7^ Neurotrophic factors such as Nerve Growth Factor (NGF), Ciliary Neurotrophic Factor (CNTF), and Transforming Growth Factor β (TGF-β1) are the most important stimulators of axon growth, and problems in regeneration are observed if these are not released at sufficient levels. The effects of PNIs can vary depending on the damage to motor, sensory, and autonomic fibers. The complex nature of the treatment of PNIs, in fact, derives from this, because irrespective of the cause, different symptoms occur in such injuries. PNIs can be associated with any combination of nociceptive, neuropathic, and avulsion pain syndromes. Peripheral nerve damage causes neuroinflammation, which plays a critical role in establishing and maintaining neuropathic pain.^8^, ^9^ Medical therapies are therefore important in addition to surgical techniques. Steroids are a good option in medical treatment. These relieve pressure on nerve tissue by reducing tissue swelling and inflammation. Steroid use is also thought to be capable of reducing secondary injury and ensuring normal homeostasis through its membrane-stabilizing effect.^10^ Steroids are widely employed in injuries to the CNS since they are thought to act as a neuroprotective agent by inhibiting neuronal apoptosis. While they were previously employed to reduce edema developing subsequently to CNS injury, they have recently been used due to such properties as inhibiting lipid peroxidation and inflammatory cytokines, preserving calcium homeostasis, restoring local blood flow, and modulating of inflammation. However, steroid therapy also entails various side effects, which restrict its therapeutic use.^11^

Pregabalin is of proven efficacy in the treatment of neuropathic pain, postherpetic neuralgia, and fibromyalgia associated with diabetic peripheral neuropathy, although there has been little investigation of its nerve regeneration and anti-inflammatory effects. Pregabalin is a Gamma-Aminobutyric Acid (GABA) analogue. However, although its chemical structure resembles that of GABA, it does not behave like GABA and does not bind to GABA receptors. It reduces depolarization-induced calcium influx following powerful binding to the alpha-2-delta (α2-δ) subunit of calcium channels in the CNS. By reducing the release of excitatory neurotransmitters, pregabalin prevents the accumulation of intracellular calcium, free radical formation, and a process that results in lipid peroxidation, thus leading to clinical improvement in nerve damage.^12^^,^^13^

Experimental studies have shown the effects of steroid or pregabalin use on peripheral nerve healing.^14^^,^^15^ However, the authors encountered no studies comparing these to agents and/or showing their effects when employed in combination. The main purpose of this study is to assess the neuroprotective, regenerative, and anti-inflammatory effects of pregabalin and methylprednisolone, used separately and in combination, and to evaluate whether pregabalin is capable of use as an alternative supportive medical treatment to steroids in cases of peripheral nerve damage.

## Material and methods

### Animals

This study was conducted at the Ataturk University Medical Experimental Application and Research Center. All experimental procedures were performed in accordance with national guidelines for the care and use of laboratory animals. Ethical approval was obtained from the local Animal Care and Use Committee (Approval n° E-75296309-050.01.04-2200083684, dated 2022–03–07), and the study adhered to the principles outlined in the Declaration of Helsinki. The experimental design also complied with the ARRIVE (Animal Research: Reporting of In Vivo Experiments) guidelines.

A total of 56 male Sprague-Dawley rats, aged 2–3 months and weighing 200–250 g, were used in this study. All animals were sourced from the university’s laboratory animal facility. Rats were housed in groups of eight per cage under standard laboratory conditions, with controlled temperature (18–21 °C), humidity (40 %–50 %), and a 12-hour light/dark cycle. Food and water were provided ad libitum. No animals were excluded from the analysis.

### Sample size

The sample size was determined using G*Power 3.1.9.2 software (Heinrich-Heine-Universitat Dusseldorf, Germany). An a priori analysis employing ANOVA (fixed-effects, special, main effects, and interactions) was carried out to compute the required sample size. The input parameters were effect size (*f* = 0.40), power (power = 0.85), and α error probability (0.05) with 7 groups and 8 rats in each group (total of 56 rats).^15^

### Experimental design

A total of 56 rats were randomized so that the average weights of all groups were close to each other. The rats were divided into seven groups of eight animals each. The experimental design of groups is shown in Table 1.

**Table 1:** Experimental desing of groups. PGB: Pregabalin, MP: Methylprednisolone, Combination: Pregabalin + Methylprednisolone.

Group	Name	Treatment	Dose and Route	n
1	Control	Sham surgery	‒	8
2	Sham	Axonotmesis	‒	8
3	PGB Low	Axonotmesis + PGB	30 mg/kg, oral	8
4	PGB High	Axonotmesis + PGB	60 mg/kg, oral	8
5	MP	Axonotmesis + MP	1 mg/kg, oral	8
6	PGB Low + MP	Axonotmesis + Combination	30 + 1 mg/kg, oral	8
7	PGB High + MP	Axonotmesis + Combination	60 + 1 mg/kg, oral	8

Groups 3, 4, 5, 6 and 7 (the treatment groups) received either pregabalin (Lyrica, Pfizer, USA) or methylprednisolone (Medrol® 4 mg tablet, Pfizer, Istanbul, Türkiye) dissolved in 1 mL of distilled water via gavage once daily for a period of seven days commencing from the day of surgery. The drug dosages employed in this study were selected in light of the findings of previous research.

### Surgical procedure

The rats were fasted for eight hours prior to surgery, and were then weighed to calculate the amount of anesthetic required. Anesthesia was performed using sodium thiopental obtained from Ibrahim Ethem ULAGAY A.S. (Istanbul, Türkiye). Anesthesia was induced through the intraperitoneal administration of 20 mg/kg thiopental and 5 % sevoflurane inhalation. The surgical procedure was performed by the same surgeon. The animals’ right gluteal and lateral femoral regions were shaved. The rats were placed in the ventral decubitus position, after which an incision approximately 3 cm in length and parallel to the right femoral region was made. The subcutaneous tissue was incised as previously described, and the sciatic nerve was exposed after gentle retraction of the biceps femoris muscle. The axonotmesis-type injury model was created through the application of a 5-cm Dietrich bulldog clamp (Tekno-Cer® AC-123–20), with a head length of 13 mm, to the sciatic nerve. This exerted a total force of 180 g (equivalent to 1.76 Newton [N]) for one minute and was then removed.^16^ Finally, the incisions were closed with 4–0 polypropylene suture (Prolene, Ethicon R Ltd, Somerville, NJ, USA). The presence of nerve injury was confirmed by means of Electromyography (EMG).

The animals were euthanized by means of high-dose thiopental (50 mg/kg) on the 28th day after the surgical procedure. Intracardiac blood samples were collected to be used in biochemical examinations, and the sciatic nerve was removed. All animals were euthanized in line with routine ethical protocols.

### Footprint analysis and functional evaluation

Sciatic Functional Index (SFI) evaluations were carried out on the day before the surgery, and on the seventh and 28th days postoperatively, using a closed racetrack measuring 8.2 × 42 × 12 cm. This involved the animals walking along the racetrack using their hind legs, which had been previously stained with blue ink.

At the end of the walking test, precise measurements were calculated based on the animals’ footprints using millimetric scales. Various parameters were assessed, including: 1) The distance between the heel and the third phalanx (print length, PL); 2) The distance between the first and fifth phalanges (toe spread, TS); 3) The distance between the second and fourth phalanges (intermediate toe spread, IT).

Measurements were determined for both the Experimental limb (E) and the contralateral, Normal limb (N).

The SFI was calculated using the formula SFI=−38.3(EPL−NPL)/NPL+109.5(ETS−NTS)/NTS+13.3(EIT−NIT)/NIT−8.8 (E: Experimental side, N: Normal side).

The evaluations were assigned values between 0 and 100. An SFI value of 100 is regarded as indicating total loss of nerve function, while a value of approximately 0 is regarded as indicative of normal nerve function.^17^

### Electrophysiological evaluation

Electromyography (EMG) was performed in order to evaluate nerve conduction following axonotmesis-type injury. These evaluations were carried out on the first day postoperatively, and also at the end of the first and fourth weeks (days 7 and 28). In order to achieve minimal disruption to the EMG recordings, thiopental was injected via the intraperitoneal route, and all rats inhaled sevoflurane prior to each EMG procedure.

A Cadwell Cascade Elite electroneuromonitorization device was employed for electrophysiological examinations. The postoperative neuro-monitoring parameters were configured in the form of a filtering range of 1‒3 k/Hz, a sweeping time of 10 ms/div, a stimulus time of 0.3 ms, and a stimulus frequency of 2.71 s. Twisted needle electrodes made of stainless steel with 13 mm-long copper wires were carefully inserted into the gastrocnemius muscle, while reference electrodes were placed in the piriformis muscle. Motor impulses were recorded by means of inserting the electrodes into muscles innervated by the relevant nerves.

It is important to note that the technicians performing the measurements were blinded to the group assignments. A number of parameters were calculated, such as the compound muscle action potentials deriving from sciatic nerve stimulation, the speed of action potential transmission between consecutive impulses, and alterations in the amplitude and area of compound action potentials. Impulse data from the experimental right leg and the reference left leg were employed to determine the experimental limb/reference limb ratio. Subsequent statistical comparisons were performed on the basis of these data.

### Histopathological evaluation

Tissue specimens collected at the end of the evaluation were fixed for 48 h in 10 % formaldehyde solution, subjected to routine fixation procedures, and embedded in paraffin blocks. Sections 4 mm in thickness were taken from each block. The preparations made ready for histopathological analysis were then stained with Hematoxylin-Eosin (HE) and examined under a light microscope (Olympus BX 51, Japan). Depending on their histopathological features, the sections were classified as none (-), very mild (+), mild (+ +), moderate (+ + +), or severe (+ + + +).

### Immunohistochemical evaluation

Prior to being mounted onto poly-l-lysine adhesive slides for immunoperoxidase analysis, tissue sections underwent deparaffinization and drying. Next, endogenous peroxidase was inactivated by immersing it in 3 % H_2_O_2_ for ten minutes. The tissues were then allowed to cool at room temperature after being cooked in a 1 % antigen retrieval (citrate buffer [pH + 6.1] 100 ×) solution. In order to prevent background staining that was not specific, the preparations were incubated with a protein block for five minutes. Following the application of primary antibodies (Nucleus Kappa B [NF-Κb] cat. no. sc - 8414, dilution ratio 1/100, USA), the tissue was incubated according to the manufacturer's guidelines. The chromogen utilized in the tissues was 3–3-'Diaminobenzidine (DAB). The slices that were stained were scrutinized using a Zeiss Axio light microscope (Germany).

### Immunofluorescence evaluation

Tissue sections were dried and deparaffinized before being adhered to poly-l-lysine adhesive slides for immunofluorescence analysis. The slices were subsequently left in 3 % H_2_O_2_ for ten minutes to inactivate the endogenous peroxidase. After boiling the tissues in 1 % antigen retrieval (pH+6.1, 100 ×) citrate buffer solution, they were allowed to cool at room temperature. In order to avoid background staining that is not specific, the sections were also allowed to incubate with protein block for five minutes. After that, the tissues were covered with the primary antibody (NGF cat. n° BS23061941, dilution ratio: 1/100, USA), which was then incubated according to the usage directions. For 45 min, secondary antibodies (FITC cat. n° ab6785, dilution ratio: 1/1000) were utilized as secondary markers for immunofluorescence and were stored in the dark. After being in the dark for five minutes, the sections were covered with glass covers. Mounting media containing DAPI (cat. n° D1306, dilution ratio: 1/200 UK) was then applied to the sections. Finally, a microscope equipped with a fluorescent attachment (Zeiss AXIO, Germany) was used to view the stained tissues.

### Biochemical evaluation

The biochemists performing the analysis were unaware of the group assignments. A total of 5 mL of intracardiac blood was collected into blood biochemistry tubes. The whole blood samples were then subjected to centrifugation at 4500 rpm for 7 min to isolate the sera, which were subsequently stored at −80 °C until the study date. Serum NGF, Ciliary Neurotrophic Factor (CNTF), Myelin Basic Protein (MBP), and Transforming Growth Factor Beta (TGF-β) values were determined using the ELISA method with a Rat NGF ELISA kit (BTLAB, cat n° E0539Ra, China), a Rat CNTF ELISA kit (BTLAB, cat n° E0358Ra, China), a Rat MBP ELISA kit (BTLAB, cat n° E0576Ra, China), and a Rat TGF-β ELISA kit (BTLAB, cat n° E0778Ra, China) in accordance with the manufacturer’s recommendations.

### Statistical analysis

Statistical Package for the Social Sciences version 25 and GraphPad Prism 8.0.2 software were employed for data analysis. The data were presented as the mean and standard deviation values. The normality of data distribution was assessed using the Kolmogorov-Smirnov test, and homogeneity of variances was confirmed with Levene's test. SFI, EMG, and histopathological data were analyzed using the Kruskal-Wallis test, followed by the Mann-Whitney *U* post hoc test because they were not normally distributed. Additionally, as the biochemical ELISA data were found to be normally distributed, differences between groups were analyzed using one-way ANOVA, followed by the Duncan test as a post hoc test.

The histopathological evaluation was conducted using a blinded pathology method, with assessments performed independently by two different pathologists. In the analysis, five regions were identified and photographed from the tissue samples of each rat. The average of the pathological findings observed in these regions was considered the representative result for each group.

It should be noted that pregabalin is a known analgesic; however, direct and indirect measures of neuropathic pain (e.g., mechanical allodynia) were unfortunately not evaluated in this model.

## Results

### Sciatic functional index findings

With the exception of the control group, the groups’ SFI values on day 7 post-injury were close to one another (*p* = 0.624), and a significant increase was observed in all groups’ SFI values on day-28 compared to the sham group (*p* < 0.001). Analysis within the groups treated with pregabalin only revealed significantly higher SFI values in the high-dose pregabalin group than in the low-dose group (*p* = 0.002).

**Fig. 1:**
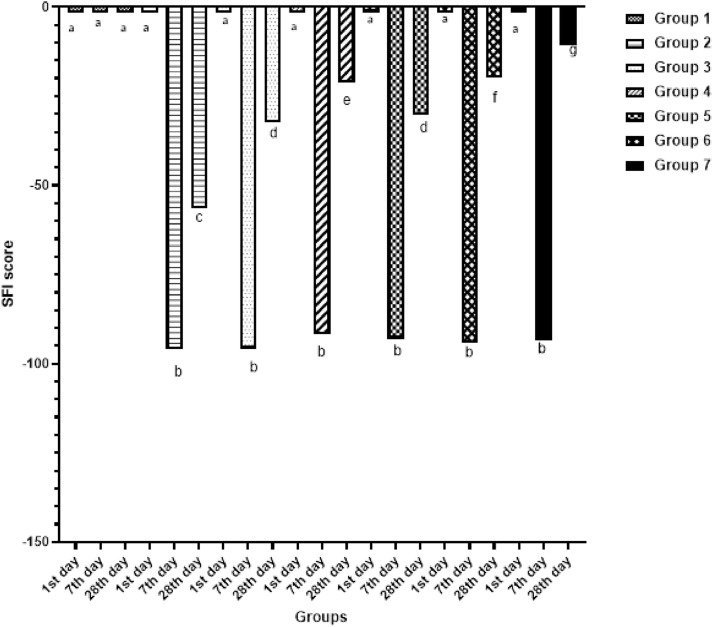
A comparison of the study groups’ SFI values on days-1, −7 and −28 post-injury. (a, b, c, d and e) There is no statistically significant difference between groups in columns symbolized by the same letter. There is a statistically significant difference between groups in columns symbolized by different letters.

SFI values in the group given steroid therapy alone were similar to those in the low-dose pregabalin group (*p* = 0.989), but significantly lower compared to the high-dose pregabalin group (*p* = 0.029). The improvement in SFI values in the groups using combined steroids and pregabalin was significantly more marked than in the groups using pregabalin only (*p* = 0.001 and *p* = 0.003, respectively) (Fig. 1).

### EMG findings

**Fig. 2:**
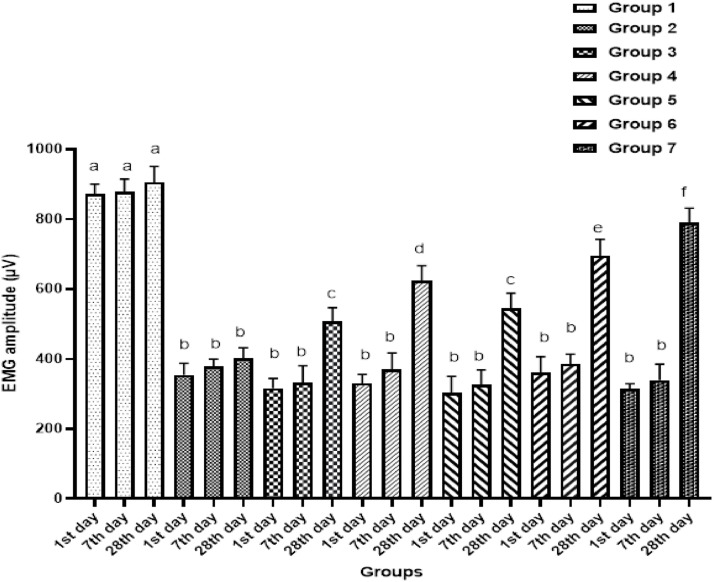
EMG findings on days-1, −7, and −28 post-injury. (a, b, c, d and e) There is no statistically significant difference between groups in columns symbolized by the same letter. There is a statistically significant difference between groups in columns symbolized by different letters. Values are means ± SD.

With the exception of the control group, the groups’ day-1 and day-7 post-injury EMG results were similar (*p* = 0.117 and *p* = 0.105, respectively). However, an improvement in EMG levels was observed in the treatment groups compared to the sham group on day 28 (*p* < 0.001 for all). EMG levels increased significantly in a dose-dependent manner in the groups treated with pregabalin (*p* < 0.001). EMG levels in the group receiving steroid therapy alone were similar to those in the low-dose pregabalin group (*p* = 0.595), but significantly lower than those in the high-dose pregabalin group (*p* = 0.019). Improvement in EMG levels was in the combined steroid and pregabalin groups was more marked than in the groups using pregabalin alone (*p* < 0.001 for all) (Fig. 2).

### Histopathological findings

**Fig. 3:**
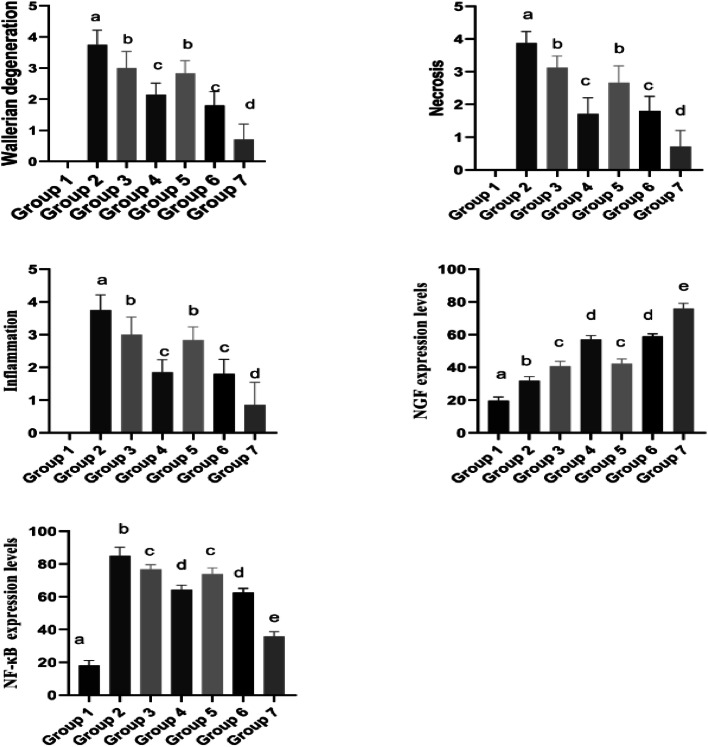
Analysis data and statistical findings concerning histopathological (Wallerian degeneration, necrosis and inflammation), immunohistochemical (NF-κB) and ımmunofluorescent examination findings (NGF) in sciatic nerve tissues. (a, b, c, d, and e) no statistically significant differences apply between groups in columns symbolized by the same letter. NF-κB, Nuclear Factor-Kappa B; NGF, Nerve Growth Factor.

**Fig. 4:**
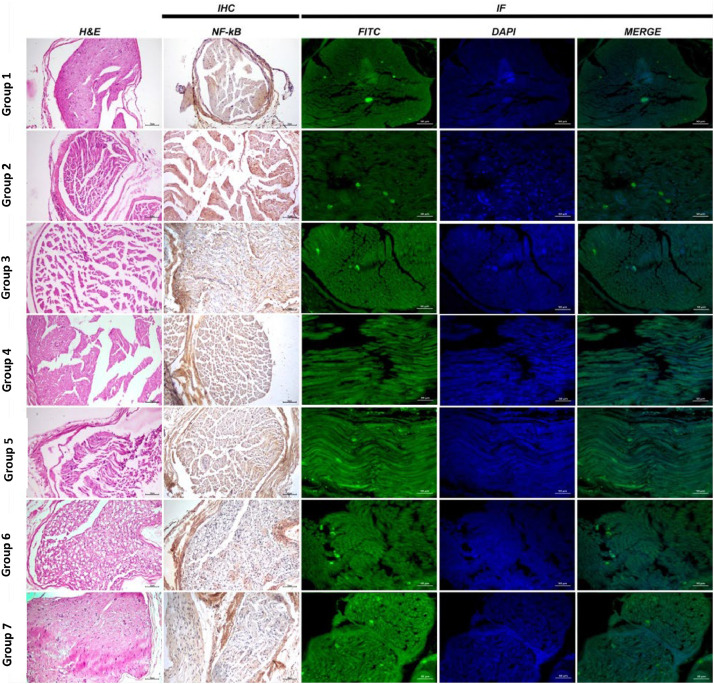
Sciatic nerve histopathological, immunohistochemical (NF-κB) and immunofluorescent (NGF) examination findings, H&E, NF-κB expression IHC-P, Bar: 70 µm, NGF expression IF, Bar: 50 µm; H&E. IHC, Immunohistochemical; IF, Immunofluorescence; NF-Κb, Nuclear Factor-Kappa B; NGF, Nerve Growth Factor.

Group 1: Sciatic nerve tissues exhibited a normal histological appearance at histopathological examination (Fig. 3, Fig. 4).

Group 2: Severe WD and necrosis in sciatic nerve tissues and inflammation and edema in interstitial spaces were observed at histopathological examination (Fig. 3, Fig. 4).

Group 3: Moderate WD, necrosis and inflammation in nerve cells were observed at histopathological examination of sciatic nerve tissues (Fig. 3, Fig. 4).

Group 4: Mild WD, necrosis and inflammation in nerve cells were observed at histopathological examination of sciatic nerve tissues (Fig. 3, Fig. 4).

Group 5: Histopathological examination of transverse sciatic nerve tissue sections revealed moderate WD, necrosis and inflammation (Fig. 3, Fig. 4).

Group 6: Histopathological examination of transverse sciatic nerve tissue sections revealed moderate WD, necrosis and inflammation in nerve cells (Fig. 3, Fig. 4).

Group 7 (Axonotmesis + Pregabalin 60 mg/kg + 1 mg/kg methylprednisolone): Histopathological examination of transverse sciatic nerve tissue sections revealed very mild WD, necrosis and inflammation in nerve cells (Fig. 3, Fig. 4).

### Immunohistochemical findings

Group 1: Nuclear Factor-Kappa B (NF-κB) expression was evaluated as negative when the sciatic nerve tissues were examined using the immunohistochemical staining method (Fig. 3, Fig. 4).

Group 2: Severe NF-κB expression was observed in the interstitial spaces around the vessels when the sciatic nerves were examined using the immunohistochemical staining method (Fig. 3, Fig. 4).

Group 3: Immunohistochemical staining revealed moderate NF-κB expression in the interstitial spaces around the vessels (Fig. 3, Fig. 4).

Group 4: Examination of the sciatic nerve using immunohistochemical staining revealed mild NF-κB expression in the interstitial spaces, around the vessels, and in the neurons (Fig. 3, Fig. 4).

Group 5: Immunohistochemical staining revealed moderate NF-κB expression in the interstitial spaces around the vessels (Fig. 3, Fig. 4).

Group 6: Examination of the sciatic nerve using immunohistochemical staining revealed mild NF-κB expression in the interstitial spaces, around the vessels, and in the neurons (Fig. 3, Fig. 4).

Group 7: Examination of the sciatic nerve using immunohistochemical staining revealed very mild NF-κB expression in the interstitial spaces, around the vessels, and in the neurons (Fig. 3, Fig. 4).

### Immunofluorescent findings

Group 1: NGF expression was evaluated as negative sciatic nerve tissue examination using the immunofluorescent staining technique (Fig. 3, Fig. 4).

Group 2: Examination of the sciatic nerve using the immunofluorescent staining technique revealed very mild NGF expression in nerve cells (Fig. 3, Fig. 4).

Group 3: Examination of the sciatic nerve using the immunofluorescent staining technique revealed a moderate level of NGF in nerve cells (Fig. 3, Fig. 4).

Group 4: Examination of the sciatic nerve using the immunofluorescent staining technique revealed intense NGF staining in nerve cells (Fig. 3, Fig. 4).

Group 5: Examination of the sciatic nerve using the immunofluorescent staining technique revealed moderate NGF expression in nerve cells (Fig. 3, Fig. 4).

Group 6: Examination of the sciatic nerve using the immunofluorescent staining technique revealed intense NGF expression in nerve cells (Fig. 3, Fig. 4).

Group 7: Examination of the sciatic nerve using the immunofluorescent staining technique revealed intense NGF expression in nerve cells (Fig. 3, Fig. 4).

### Biochemical findings

**Fig. 5:**
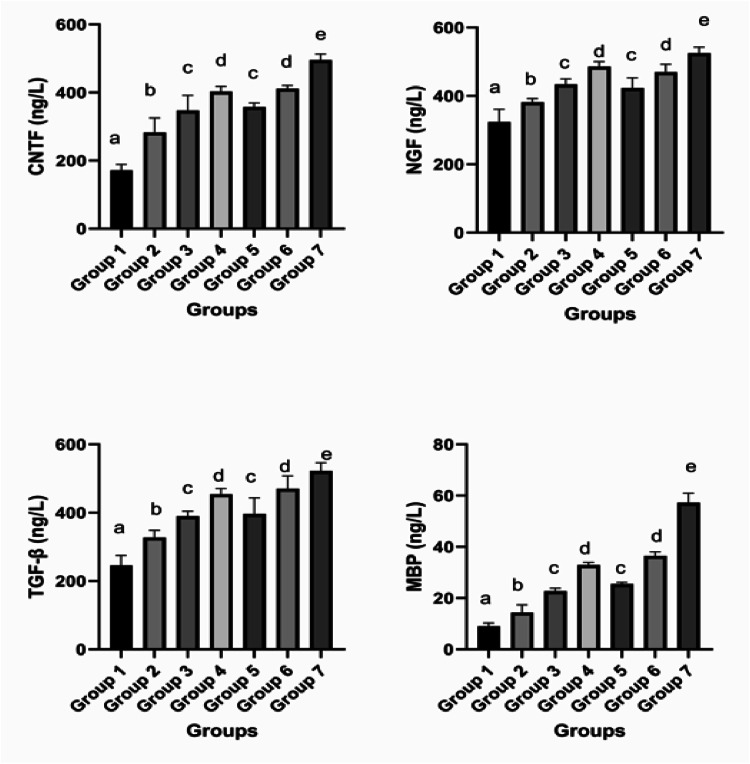
Evaluation of the groups’ ELISA results. (a, b, c, d and e) There is no statistically significant difference between groups in columns symbolized by the same letter. There is a statistically significant difference between groups in columns symbolized by different letters. CNTF, Ciliary Neurotrophic Factor; NGF, Nerve Growth Factor; TGF-β1, Transforming Growth Factor β; MBP, Myelin Basic Protein.

It is important to note that the biochemists performing the measurements were blinded to the group assignments. Serum NGF, MBP, CNTF, and TGF-β levels rose significantly following PNI compared to the control group (*p* < 0.001 for all). Marker levels were particularly higher in the treatment groups than in the non-treated sham group (*p* < 0.001 for all). The serum NGF, MBP, CNTF, and TGF-β levels in the groups treated with pregabalin rose significantly in a dose-dependent manner (*p* < 0.001, *p* < 0.001, *p* = 0.007, and *p* = 0.001, respectively). Marker levels in the group given steroid only were similar to those of the low-dose pregabalin group, but significantly lower than those of the high-dose pregabalin group (*p* = 0.228 and *p* = 0.008, respectively, for NGF, *p* = 0.210 and *p* < 0.001 for MBP, *p* = 0.992 and *p* = 007 for CNTF, and *p* = 0.999 and *p* = 0.001 for TGF-β) (Fig. 5).

## Discussion

PNI is of interest to researchers due to the high prevalence of such injuries, and increasing numbers of studies are being conducted in the field of nerve regeneration. The healing process may be prolonged in the absence of therapeutic interventions, and the injury may lead to the denervation and atrophy of associated muscles. It is therefore important to accelerate nerve regeneration in order to achieve perfect recovery.^18^^,^^19^ The results of the present study showed that methylprednisolone and pregabalin exhibited similar effects when administered separately in an experimental sciatic nerve crush injury model in rats, while combined administration was more effective in terms of functional and histomorphological healing. The authors think that pregabalin may represent an alternative medical therapy to methylprednisolone.

Early surgery for the repair of nerve injuries significantly reduces potential pain and narcotic use and shortens the rehabilitation period. Agents capable of treating nerve injuries and improving sensory and motor functions are needed. Numerous pharmacological therapies have been tried to date, although there is still no standard pharmacological therapy. Surgical interventions may be required in all cases in which spontaneous healing is not observed, although complete functional improvement may only be achieved in a very small proportion of such patients.^20^

In this study, the authors applied an experimental axonotmesis model to evaluate the effects of pregabalin on nerve regeneration. Pregabalin (S-[+]−3-isobutylgaba) was developed as a lipophilic GABA analog; However, it does not exert any direct effects on GABA-like mechanisms. Pregabalin strongly attaches to the α2-δ subunit and reducing calcium influx at nerve terminals, thus diminishing the release of various neurotransmitters such as glutamate, noradrenaline, serotonin, dopamine, and substance P. Ultimately, pregabalin improves nerve damage clinically by lowering the release of excitatory these neurotransmitters, which in turn reduces intracellular calcium buildup, the production of free radicals, and a process that leads to lipid peroxidation.^12^

When establishing such a model, it should not be forgotten that nerve damage depends on the force and duration of the crush. Various nerve damage induction techniques and evaluation tools can be employed, and this makes standardization problematic in studies. In order to achieve standardization, the authors applied 1.76 N nerve compression using a Dietrich bulldog clamp for 1-minute, and assessed the damage occurring using EMG measurements. Complete function loss was observed in all the rats in which the authors induced axonotmesis injury. This electrophysiological method is an indirect means of determining motor and sensory function and estimating nerve regeneration.

The principal aim in peripheral nerve regeneration is, of course, functional recovery. Analyzing a mouse’s walking pattern by recording its footprints is one widely employed method for assessing such recovery.^18^ Varejão et al. determined complete improvement in SFI values at the eighth week following axonotmesis induction. Similarly, SFI decreased in crushed animals, but returned to almost normal after 36 days.^21^ The SFI measurements performed on the seventh and 28th days of injury and the EMG measurements taken on the first, seventh, and 28th days in the present research clearly show that functional improvement was achieved in the treatment groups, together with the efficacy of the treatments applied. Methylprednisolone, the first agent the authors used in treatment, is an anti-inflammatory agent with proven neuroprotective efficacy tested in controlled, multi-center studies. Several studies have shown that it prevents free radical production and helps improve neurological function following spinal injury.^22^^,^^23^ Interestingly, the neurodegenerative process itself has also been shown to affect the levels of neuroactive steroids in peripheral nerves in some experimental models.^24^^,^^25^ Roglio et al. thought that axonotmesis injury reduces neuroactive steroid levels axonotmesis, and that the administration of steroids such as dihydroprogesterone and progesterone is a promising approach in terms of accelerating healing at the biochemical, morphological, and functional levels.^14^ However, despite their beneficial effects, the long-term systemic use of these agents is also known to lead to osteoporosis, ophthalmological damage, adrenal suppression, hyperglycemia and diabetes, cardiovascular disease, dyslipidemia, dermatological and gastrointestinal events, psychiatric disorders, and immunosuppression.^26^

Few experimental studies have examined the effects of pregabalin on nerve regeneration. Whitlock et al. suggested that pregabalin does not affect peripheral nerve regeneration following crush injury based on SFI and histomorphological examinations.^27^ In contrast to that study, Çelik et al. examined the effects of two different doses of pregabalin, 30 mg/kg and 60 mg/kg, on nerve healing, and found that the 30 mg/kg dose was more effective in improving nerve regeneration and increasing SFI.^15^ In their experimental study, Çalıkoğlu et al. showed that pregabalin exhibits anti-edematous, anti-inflammatory, and neuroprotective effects in traumatic brain injury.^28^ Another problem closely associated with nerve damage or inflammation is neuropathic pain. Pregabalin is one of the most effective pharmacotherapeutic treatments for neuropathic pain control. Rodrigues et al. showed the effect of pregabalin on neuropathic pain in a sciatic nerve chronic constriction injury model in rats.^29^

Neurotrophic factors are a family of polypeptides that neurons require for survival and that represent the most important stimulators of axon growth and regeneration. NGF, evaluated by means of biochemical examination in the present study, is a molecule essential for growth and healing that affects both motor and sensory neurons. CNTF is a member of the IL-6 family with important neuroprotective effects on neurons. It enhances the rate of axonal elongation, axonal sprouting, and reinnervated muscle end plate numbers and accelerates functional recovery. Chen et al. showed that the combination application of NGF and CNTF may help significantly improve the functional recovery of the sciatic nerve.^30^ Sencar et al. found that the combined use of NGF and betamethasone increased axon numbers and myelin thickness following PNI. They also proposed that the neuroregenerative effects of NGF and the anti-inflammatory and/or antiedematous effects of betamethasone further enhanced functional recovery.^23^

TGF-β is of crucial importance in numerous physiological processes, such as the immune response, cellular proliferation and apoptosis, cellular adhesion and migration, and wound healing. It is essential for nervous system development and exhibits known neuroprotective effects, regulating the behaviors of neurons and glial cells following nervous system damage and thus mediating the regenerative process.^31^^,^^32^ MBP is the second most abundant protein in the CNS. It is a structural protein essential for the formation of CNS myelin, and is also known as the ‘executive molecule of myelin’.^33^ Lutz et al. determined that MBP improves healing following acute nerve injury.^34^ TGF-β values in this research were lower, and MBP values were higher after treatment, with the exception of the sham group. This shows that methylprednisolone and pregabalin are effective in reducing inflammation, increasing MBP expression in the damaged axon, and promoting nerve regeneration. Serum NGF, CNTF, MBP, and TGF-β levels in the groups in which pregabalin and methylprednisolone were combined were closer to those of the control group than in the other groups suggesting that the combined use of these drugs can be more effective in nerve regeneration and functional recovery.

The principal objectives are the elimination of structural changes in the injured nerve, restoration of a normal structure, and thus functional recovery. Since inflammatory reactions developing following PNI can lead to severe structural changes in cells and tissues, inflammatory reactions must be closely monitored in the evaluation of nerve regeneration. Through their anti-inflammatory and antiedematous effects, steroids reduce fibrosis and edema in the lesion region. Ha et al. showed that pregabalin can behave as a neuroprotector, and that its anti-apoptotic and anti-inflammatory effects are associated with its neuroprotective activity.^35^ Similarly, in the present study, histopathological examination showed that pregabalin and methylprednisolone reduced inflammation, necrosis, and WD levels on the 28th day. This may be attributable to more rapid regeneration in the treatment groups, especially in the high-dose combination.

Studies have suggested that NF-κB expression in PNI suppresses the inflammatory response and delays nerve regeneration. In contrast, suppressing NF-κB pathway activation reduces SC losses and thus creates a neuroprotective effect that alleviates sensory and motor dysfunctions associated with PNI. Dexamethasone is an NF-κB inhibitor and an anti-inflammatory glucocorticoid frequently used to treat nerve inflammation after injury.^36^^,^^37^ In this study, immunohistochemical and immunofluorescent findings again show that NF-κB expression decreases and NGF expression increases in low-dose and high-dose treatment combinations and that regeneration is more rapid, especially in these groups.

Neuropathic pain is a major clinical challenge, which is frequently associated with peripheral nerve injury or inflammation and can have a serious impact on a patient quality of life. Following nerve injury, infiltrating leukocytes secrete abundant proinflammatory cytokines to directly sensitize nociceptors, leading to the induction of neuropathic pain. Pregabalin is one of the most popular analgesics used for neuropathic pain relief. The effects of pregabalin have been documented in various pain models in previous studies.^38^^,^^39^ In this study, the authors think that neuropathic pain due to PNI in rats was also reduced by pregabalin.

The lack of direct neuropathic pain assessment in animals is a significant limitation, given pregabalin's primary use for this condition. Another limitation is the lack of a direct behavioral or nociptive assessment of neuropathic pain, despite the analgesic role of pregabalin. The translation of findings from rat models to clinical practice can create restrictions. Pregabalin may represent a potential alternative or adjunct to steroids in peripheral nerve injuries, but further preclinical and clinical studies are required to confirm these findings.

In conclusion, the combined use of pregabalin and methylprednisolone is more effective than their separate use. In combined use, neuroprotective, regenerative, and anti-inflammatory effects are more pronounced in peripheral nerve damage. Findings on the efficacy of pregabalin-methylprednisolone combination therapy could influence future research and treatment protocols. Therefore, pregabalin may represent a viable alternative for patients in whom corticosteroid therapy is contraindicated, and its adjunctive use with steroids could lead to improved treatment protocols.

## Ethics approval and consent to participate

Ethical approval for this study was granted by the Ataturk University local ethics committee (n° E-75296309–050.01.04-2200083684) on 2022–03–07 and in accordance with the tenets of the Declaration of Helsinki.

## Consent for publication

Not applicable.

## Authors’ contributions

Methodology, İ.A., E.L., S.Y., A.A.; Investigation, S.Ö., B.P., D.K. and İ.B.; Resources, E.L., B.I. and T.Y.; Writing-original draft, İ.A., A.K.; Writing-review and editing, İ.A., B.I., N.K. and E.L.; Supervision, N.K., M.S.C.; Formal analysis, B.I., A.K., A.A.; Data curation, A.A., İ.B.; Software, A.A., İ.B.; Project administration, İ.A. All authors have read and agreed to the published version of the manuscript.

## Funding

This research was funded by the Researchers Supporting Project code n° TAB-2022–10360, Ataturk University.

## Data availability statement

The datasets generated and/or analyzed during the current study are available from the corresponding author upon reasonable request.

## Conflicts of interest

The authors declare no conflicts of interest.

## Appendix Supplementary materials

Image, application 1
