# Supplementary material for: Pregabalin as a neuroprotective alternative to methylprednisolone in peripheral nerve regeneration: an experimental study
Source: Clinics (Sao Paulo). 2025 Nov 11;80:100838. doi: 10.1016/j.clinsp.2025.100838 (PMC12657751; doi:10.1016/j.clinsp.2025.100838)
Supplement: Supplementary file 1 [file mmc1.pdf]

4th June, 2025

Sancak Mahallesi,

Kahire Caddesi, 539. Sok.

Mavi Köşk Ap. 5/5,

Çankaya,

Ankara,

Turkey

To whom it may concern;

I am pleased to confirm that I have edited the article titled ‘Can pregabalin represent an alternative to steroids in peripheral nerve recovery? An experimental study‘ and assume responsibility for its English language content.

I am a native speaker of English and have been actively engaged in this field for the last 20 years.

Yours faithfully

Carl N. Rossini

carlninorossini@yahoo.com
